# Thermal Analysis and SEM Microscopy Applied to Studying the Efficiency of Ionic Liquid Immobilization on Solid Supports

**DOI:** 10.3390/ma12101579

**Published:** 2019-05-14

**Authors:** Anna Sowińska, Magdalena Maciejewska, Laina Guo, Etienne Delebecq

**Affiliations:** 1 Institute of Polymer and Dye Technology, Lodz University of Technology, Stefanowskiego Street 12/16, 90-924 Lodz, Poland; magdalena.maciejewska@p.lodz.pl; 2 Hutchinson S.A-Research & Innovation Center, Rue Gustave Nourry BP31, 45120 Châlette sur Loing, France; laina.guo@hutchinson.com (L.G.); etienne.delebecq@hutchinson.com (E.D.)

**Keywords:** ionic liquids, solid supports, thermogravimetry, DSC, fillers, immobilization

## Abstract

Ionic liquids (ILs) are widely used in elastomer composites, primarily as vulcanization activators or accelerators, crosslinkers, conductive additives, or dispersing agents of fillers. The aim of this work was to study the efficiency of ionic liquid immobilization on filler surfaces using different techniques of thermal analysis and scanning electron microscopy (SEM). Ionic liquid, such as 1-decyl 3-methylimidazolium bromide (DmiBr) was grafted on the surface of silica, calcium oxide, and carbon black to improve the dispersion degree of their particles in the elastomeric matrix. Thermal analysis and SEM microscopy revealed a key role in determining the efficiency of the filler modification with ILs dissolved in acetone. Identifying the weight loss associated with thermal decomposition of DmiBr in modified fillers, allowed the calculation of the efficiency of their modification and compare the surface reactivity of studied fillers with DmiBr. Silica and carbon black exhibited high and comparable ability for interaction with ionic liquid. SEM images showed that particles of DmiBr-modified fillers were quite homogeneously dispersed in the elastomer matrix and exhibited good adhesion to the elastomer.

## 1. Introduction

Rubbers, in general, are used with a number of additives, such as curatives, anti-aging substances, dyes or moisture absorbers. However, one of the most important components of rubber compounds are fillers, especially active ones, that increase hardness and modulus of vulcanizates, improve processability of rubber compounds and the mechanical properties of elastomers [1,2,3,4]. Some of the fillers give the rubbers special properties, such as lower flammability, increased thermal resistance, thermal conductivity, high or low coefficient of friction, and shape durability [5,6]. Filled elastomers are widely used engineering materials. Their main applications are automotive (tires and rubber accessories), aerospace or machine industry, but they are also used in the textile and even chemical, pharmaceutical or food industries [7,8]. Fillers can be inorganic as well as organic materials with varying degrees of fineness, and they are usually classified into three main groups, i.e., active, semi-active, and non-active. Active fillers are the most useful, and some of the most popular are silica and carbon black [9,10,11], but their surface chemistries are very different [12]. Several factors, such as specific surface area, particle size, and shape of the filler, influence their reinforcing effect [13,14]. However, no less important is their appropriate dispersion in the elastomeric matrix [15], the structure or morphology of the filler and its surface activity [16]. Filler-rubber interaction plays a crucial role in the reinforcement of rubber, whereas filler–filler interaction is generally detected by the Payne effect in dynamic measurements [17]. A decrease in filler networking improves filler-rubber interaction since strong interactions between filler particles result in a high degree of agglomeration and, therefore, poor contact between these particles and elastomer chains. Moreover, agglomerates act as centers of stress concentration when the sample undergoes external deformations, initiating its rupture. Therefore, a suitable modification or compatibilization of the filler surface is very important from a technological point of view.

Although silica and carbon black have been used for many years in filled rubber products, ensuring homogeneous dispersion of their particles in the elastomer remains a technological challenge, particularly for nanosized fillers. To select an appropriate dispersing agent, the chemical nature of the filler must be considered, so the functional groups on its surface should be taken into account.

Carbon black (CB) is the most widely used filler, especially in the tire industry, due to both a filling and reinforcing effect [18,19]. Generally, carbon black consists of aggregates of coalesced, fused elementary particles with dimensions of 10–500 nm, which tend to agglomerate into larger units. The surface of CB is usually compared to degraded polycyclic aromatic hydrocarbons with groups of varying degrees of oxidation [20]. These groups may have an acceptor character (e.g., carboxylic, carbonyl, anhydride, lactone, lactol, or hydroxyl groups) as well as a donor (e.g., γ-pyrone structure). Currently, it is assumed that oxygen groups do not play a significant role in reinforcing of most elastomers; more important are hydrogen atoms present on the edge of the CB graphite surfaces [21]. Silica has been used in the tire industry, due to its low rolling resistance and ice grip, and great wet traction. Silanol groups on the silica surface cause strong interaction between its particles, which results in a strong agglomeration leading to poor dispersion of the filler in the elastomer matrix and poor processability of rubber compounds [22,23]. Owing to the modification of the silica surface, the structure of its aggregates in rubbers can be tailored. This increases the interaction of silica with rubbers and improves the dispersion of fillers [24,25]. Another approach to improving the dispersion degree of silica particles in the elastomer is using silica coupling agents, which enhance the interaction between elastomer chains and particles of silica [26]. Regarding silica, commonly used coupling agents are silanes, whereas nitrosamines are applied as promoters of CB adhesion to rubbers [17,27,28,29,30].

In the rubber industry, calcium oxide (CaO) plays an important role as a moisture absorber. It exhibits hygroscopic properties and absorbs 32% of water in relation to its mass; during heating, it is released to form calcium hydroxide [31], which is stable at the temperatures used in the vulcanization process. The use of powdered calcium oxide is extremely difficult, due to its strong water absorption, particularly during storage. Therefore, it is difficult to obtain a homogeneous dispersion of its particles in the elastomer [32].

In the last few years, applying ionic liquids (ILs) to improve the dispersion degree of fillers in the elastomeric matrix has increasingly being reported, particularly for alkylimidazolium ILs. Owing to their non-flammability, non-volatility, and good thermal stability, ILs can be successfully used in the elastomer composites [33,34]. The chemical structure of ILs promotes the dispersion of nanoparticles in rubbers, due to the surface modification of the nanoparticles with the ILs, particularly in the case of silica, clays, carbon black, and carbon nanotubes [35,36,37,38]. Attractive interactions between the imidazolium cation of the ionic liquid and the π-electrons on the CB surface were reported to improve the dispersion degree of CB particles in elastomers [39]. Regarding silica, the strong interactions were partially attributed to hydrogen bonding between the imidazolium cation of the IL and the Si-O-Si groups of silica and partially to hydrogen bonding between the anion of the IL and the Si-OH groups on the silica’s surface [40]. 

A recent concept is the supported ionic liquid phase materials (SILPs), where a thin film of IL is immobilized on a solid phase, combining the advantage of ILs with those of heterogeneous support materials. SILP materials could be prepared using supports with different porosity and chemical nature [41]. Immobilization or supporting of ILs can be performed by different methods: simple impregnation, grafting, polymerization, sol-gel, encapsulation or pore trapping [42]. In recent years, SILP materials have been widely applied as very effective heterogeneous catalytic systems in organic reactions, such as: Hydroformylation of 1-butene [43], continuous methanol carbonylation [44], oligomerisation of isobutene [45], Heck coupling [46], or Suzuki reaction [47]. The main advantages of these catalytic systems are their efficient recycling and reusability. Most of the SILP materials are based on the ILs immobilized on the surface of silica, silica gel, or mesoporous silica [48] but layered clays [49], zeolites [50], or magnetic nanoparticles [51] can also be successfully used as solid supports for ILs immobilization.

Regarding elastomer composites, ILs are usually mixed directly with the rubber during the preparation of rubber compounds. The aim of this study was to introduce the ionic liquid into the rubber in the form of SILP, in order to improve the dispersion of filler particles and to control the IL activity in the vulcanization process. We applied 1-decyl-3-methylimidazolium bromide (DmiBr) to modify the surface of silica, CB and CaO in solvent (acetone) using ultrasonic treatment. Regarding the improvement of filler particles dispersion in the elastomer, we expected that this type of modification should be more efficient than direct mixing of IL and filler with elastomer, during the preparation of rubber compounds, using a two-roll mill. Moreover, our previous research has shown that the disadvantage of applying ILs, introduced directly into the rubber, is the significant shortening of the scorch time, which prevents the safe processing of rubber composites [52,53]. Therefore, IL was immobilized on the filler surface to reduce its activity in the temperature of processing of rubber compounds. Thermal analysis techniques, such as Thermogravimetry with Mass Spectrometry (TG-MS) and Differential Scanning Calorimetry (DSC), were employed to study the efficiency of filler modification with DmiBr, thermal stability of modified powders, and the temperature of DmiBr release from the surface of modified fillers. Scanning Electron Microscopy (SEM) was used to study the effect of filler surface modification with DmiBr on the size and morphology of their particles and their dispersion in the elastomeric matrix. 

## 2. Materials and Methods

### 2.1. Materials

**Scheme 1:**
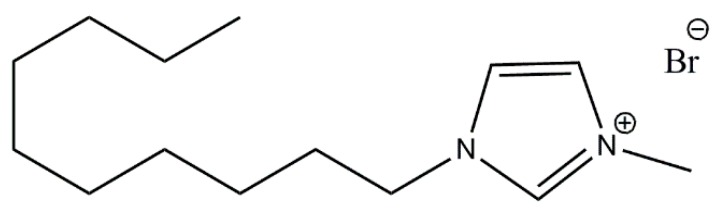
Structure of DmiBr.

DmiBr (purity >98%) with the structure shown in Scheme 1 was manufactured by IoLiTec Ionic Liquids Technologies GmbH (Heilbronn, Germany). Two types of silica were studied: Ultrasil VN3 with specific surface area 180 m^2^/g, purity >97% (manufactured by Evonik Industries, Essen, Germany) and silicon dioxide nanopowder with particle size 10–20 nm (BET), purity 99.5% (manufactured by Sigma-Aldrich, Schnelldorf, Germany). Carbon black Spheron SOA was provided by the Cabot Corporation (Boston, MA, USA). Calcium oxide nanopowder with a particle size of about 160 nm (BET) and purity 98% was manufactured by Sigma-Aldrich, Schnelldorf, Germany. Acetone used as a solvent for filler modification was delivered by Avantor Performance Materials S.A., Gliwice, Poland.

### 2.2. Modification of Fillers Surface with DmiBr

Two modifications were performed for each filler, using 10 and 20 wt% of IL, relative to the weight of the filler. Acetone was used as a solvent for DmiBr, since it was reported to dissolve most of the hydrophilic and hydrophobic ILs, due to hydrogen-bonding interactions [54]. A weighed portion of DmiBr was dissolved in acetone (300 ml) in the round bottom flask. Next, the filler was added (20 g) to form a cloudy suspension. The prepared mixture was placed in the SONOREX ultrasonic bath for 15 minutes (BANDELIN electronic, Berlin, Germany). Then, the mixture was left overnight for sedimentation. Evaporation of acetone was performed the next day with a rotary vacuum evaporator (BUCHI Labortechnik AG, Flawil, Switzerland). Before the evaporation process, a part of acetone was removed from the flask by decantation to reduce the time of evaporation. Next, the content of the flask was mixed for 10 min and the remaining acetone was evaporated. Parameters of the evaporation process: characteristic pressure for acetone: 560 mbar (for temperature 40 °C), set temperature: 40 °C, vapour temperature: 24 °C, rotation: 80 rpm. After half an hour, the pressure was reduced to 450 mbar at the beginning of solvent evaporation. The duration of the evaporation process was 250 min. DmiBr-modified fillers were inserted into the vacuum dryer (Memmert, Schwabach, Germany) overnight (50 °C, 50 mbar), and the second step was drying them in the dryer chamber at 70 °C for 3 days. Received powders were marked as follows: VN3/IL10, VN3/IL20 (Ultrasil VN3 silica modified with 10 wt.% or 20 wt.% of the IL, respectively), nanoSiO_2_/IL10, nanoSiO_2_/IL20 (silicon dioxide nanopowder modified with 10 wt.%, or 20 wt.% of the IL, respectively), CB/IL10, CB/IL20 (carbon black modified with 10 wt.%, or 20 wt.% of the IL, respectively).

### 2.3. Characterization of Pure and Modified Fillers

Thermogravimetric measurements were carried out using thermogravimetry/differential scanning calorimetry TGA/DSC1 analyzer (Mettler Toledo, Greifensee, Switzerland) in the temperature range of 25–600 °C, with a heating rate of 10 °C/min in an argon atmosphere (flow rate 50 ml/min.). Prior to the measurements, Thermogravimetry (TG) analyzer was calibrated using indium and zinc as standards. Additional analysis was performed using Setsys TG-DTA 16/18 analyser (SETARAM Instrumentation, Caluire-et-Cuire, France) coupled to a Balzers (Pfeiffer) mass spectrometer for evolved gas analysis.

DSC1 analyzer (Mettler Toledo, Greifensee, Switzerland), calibrated with indium and n-octane as standards, was employed to study thermal transitions of pure and DmiBr-modified fillers and the temperature of ionic liquid release/desorption from the surface of filler. The measurements were performed in the temperature range of 25–500 °C, with a heating rate 5 °C /min.

Rubber compounds of ethylene-propylene-diene elastomer (EPDM, Vistalon 8600, Exxon Mobile, Irving, TX, USA) containing 20 phr of DmiBr-modified fillers were prepared using a laboratory two-roll mill. Then, the prepared EPDM compounds were cured at 150 °C using an electrically heated hydraulic press for the optimal vulcanization time, which was determined with rotorless D-RPA 3000 rheometer (MonTech, Buchen, Germany). 

SEM images of analyzed filler surface and fractures of EPDM vulcanizates were taken using an LEO1450 SEM microscope (Carl Zeiss AG, Oberkochen, Germany). Prior to the measurement, vulcanizates were broken down using liquid nitrogen; their fractures were coated with carbon and next examined. Based on the SEM images, the morphology and size of filler particles were studied, as well as their dispersion in the elastomer matrix. Energy-dispersive X-ray spectroscopy (EDS) was used to confirm the presence of DmiBr on the surface of modified fillers. Samples of pure fillers were coated with carbon to improve the quality of SEM/EDS results.

## 3. Results and Discussion

### 3.1. Thermogravimetric Analysis (TG)

**Figure 1:**
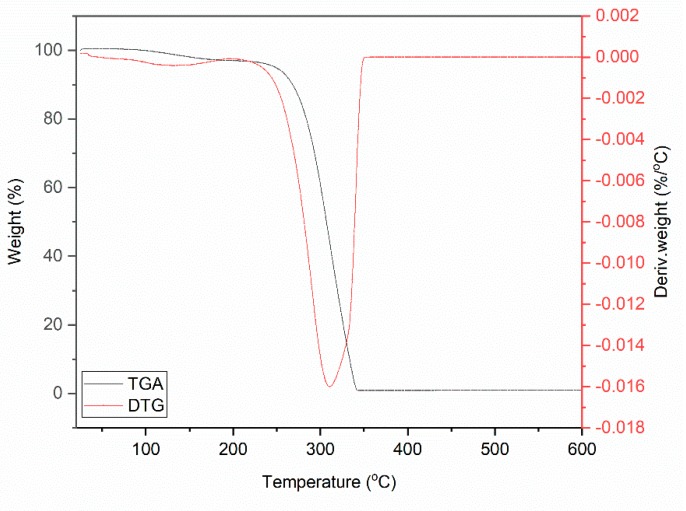
Thermogravimetric **(**TG) curve for DmiBr.

**Figure 2:**
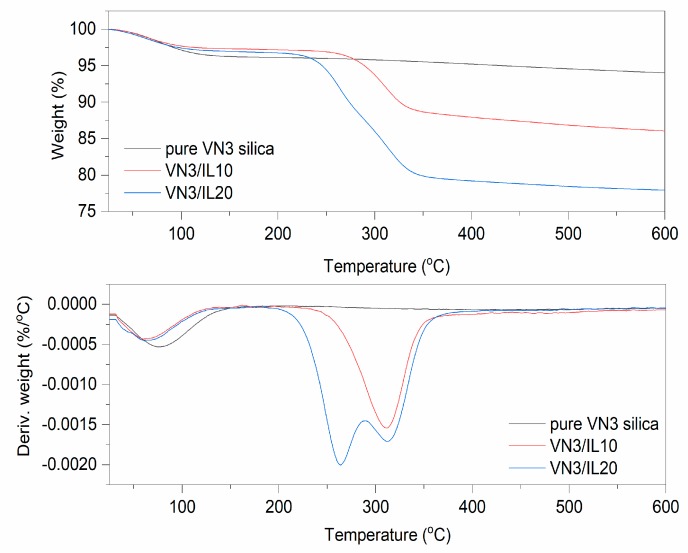
TG and Differential Thermogravimetric (DTG) curves for pure and DmiBr-modified silica VN3.

**Figure 3:**
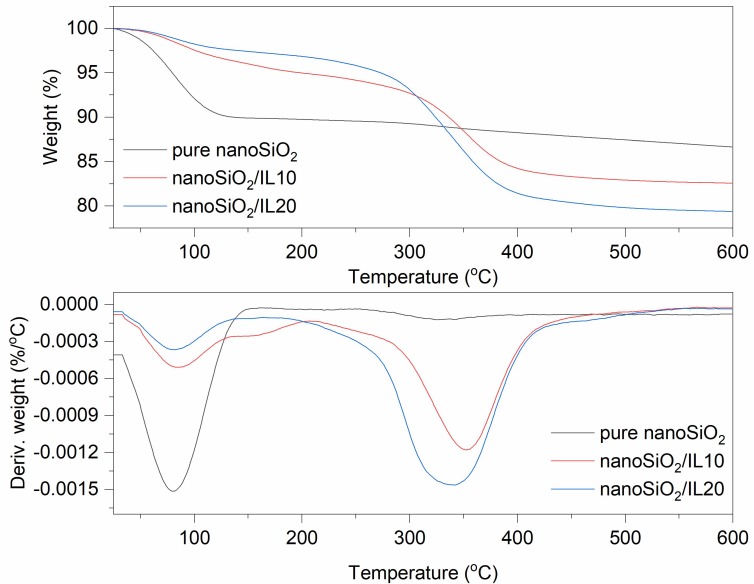
TG and DTG curves for pure and DmiBr-modified nanoSiO_2_.

**Figure 4:**
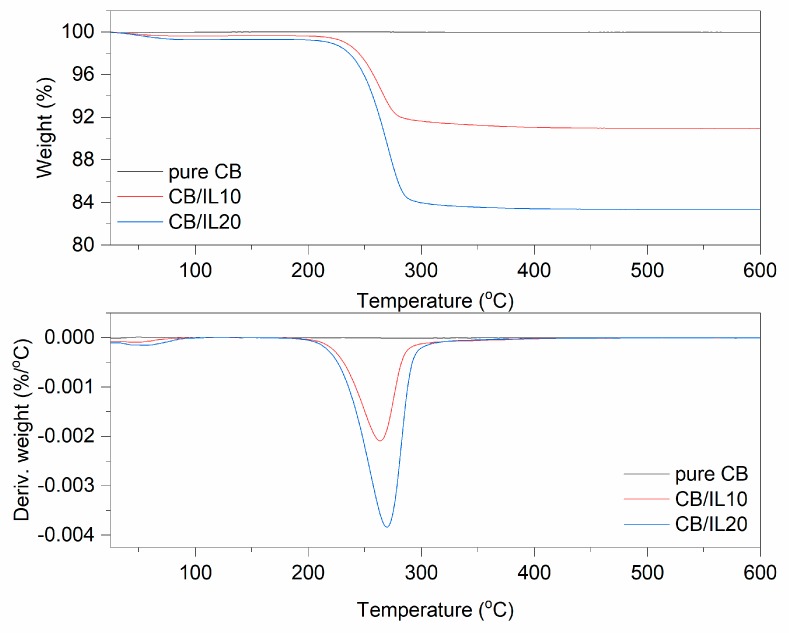
TG and DTG curves for pure and DmiBr-modified CB.

**Figure 5:**
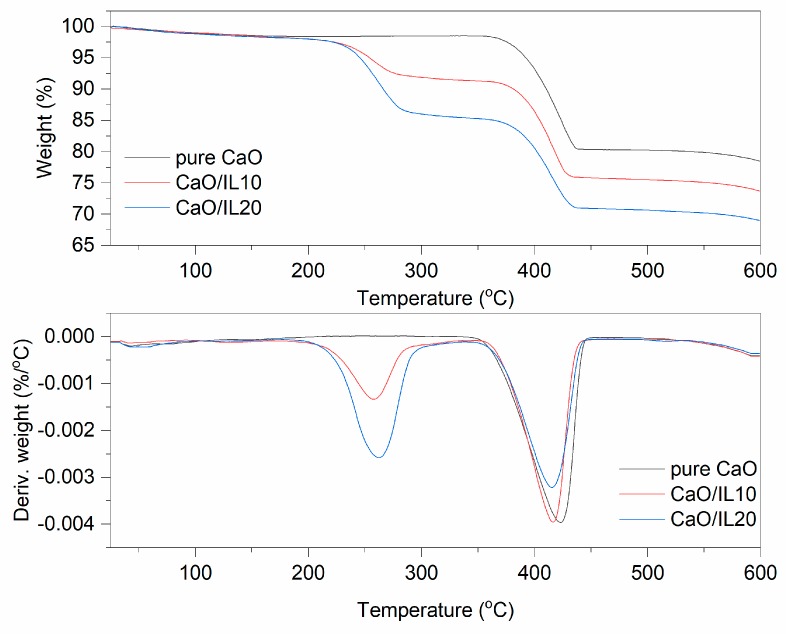
TG and DTG curves for pure and DmiBr-modified nanosized CaO.

TG was performed, in order to characterize DmiBr, as well as pure and DmiBr-modified fillers. Based on the obtained weight losses for pure and DmiBr-modified fillers, the amount of DmiBr grafted on the filler surface was determined and used to calculate the efficiency of filler modification. The results are presented in Figure 1, Figure 2, Figure 3, Figure 4 and Figure 5 and summarized in Table 1. To confirm the presence of DmiBr in modified fillers and to predict the mechanism of ionic liquid thermal decomposition, TG coupled with gas analysis (TG-MS) was carried out (Table 2 and Table 4).

Thermal decomposition of DmiBr involved two steps. The first weight loss of approximately 3.5%, in the temperature range of 50–180 °C, was likely associated with a partial desorption of moisture, whereas the main thermal decomposition of DmiBr proceeded in the temperature range of 180–400 °C with a weight loss of 96.1%. It started with the decomposition of the dodecyl chain of DmiBr into short C_x_H_y_^+^ (e.g., m/z 12, 13, 14, 15, 26, 27, 41, 42, 43, 56) fragments accompanied by the departure of bromide anion (m/z 79–81) and decomposition of the imidazolium ring of ionic liquid into different C_x_H_y_N_z_^+^ fragments, as was confirmed by TG-MS analysis (e.g., m/z 51, 52, 55, 56) (Table 2) [55]. The structural formula of DmiBr (Schema 1) was taken into account to predict the course of DmiBr thermal decomposition.

On the TG curve corresponding to the thermal decomposition of pure VN3 silica, weight loss of approximately 3.7% occurred at a temperature below 150 °C (Figure 2). This was due to the desorption of moisture, as silica is a highly hygroscopic powder. The next weight loss obtained for this filler was approximately 2.2% in the temperature range of 350–600 °C and resulted from dehydroxylation of the silica surface [56]. In the TG curve corresponding to the thermal decomposition of VN3/IL10 powder, a weight loss of approximately 5.3%, at a temperature below 150 °C was observed, which was slightly higher than that of pure silica. This may result from desorption of moisture together with solvent, which remained in this powder after silica modification with DmiBr. The weight loss of approximately 10.7% in the temperature range of 150–550 °C was associated with thermal decomposition of the ionic liquid, as was confirmed by MS analysis of the evolved gases (Table 3). Mass/charge ratios, characteristic of DmiBr decomposition, were identified in the MS spectra of VN3/IL10 in this temperature range and confirmed the presence of DmiBr in modified powder. Apart from the m/z ratios presented in Table 2, additional bands in mass spectra of DmiBr-modified fillers were achieved for the m/z assigned to ions from decomposition of DmiBr: 29-C_2_H5^+^, 31-CH_3_NH_2_^+^, 44-CO_2_, 72-C_3_H_7_CHNH_2_^+^) [55] (Table 3). 

In the case of silica VN3/IL20, the weight loss in the temperature below 150 °C (about 2.5%) was smaller than that for other samples. According to MS spectra, the weight loss of 17.9% in the temperature range of 150–550 °C corresponded to decomposition of DmiBr. Based on the weight loss associated with the decomposition of DmiBr for modified filler, and the reduction in its value, as a result of the weight loss of pure silica in the same temperature range, the actual content of ionic liquid on the silica’s surface was calculated as 8.5 and 16% for VN3/IL10, and VN3/IL20, respectively. Therefore, the efficiency of VN3 silica modification with DmiBr was 85% (VN3/IL10) and 79% (VN3/IL20) taking into account the initial amount of DmiBr, which has been used for silica modification (Table 3).

Thermal decomposition of pure nanoSiO_2_ proceeded with desorption of water at a temperature below 150 °C, with weight loss of approximately 10% (Figure 3). It can be concluded that nanosized silica is more hygroscopic than VN3. At higher temperatures, an additional 3.2% of the filler decomposed with dehydroxylation of the nanoSiO_2_ surface. The weight loss in the temperature range of 30–150 °C for nanoSiO_2_ modified with 10% of DmiBr was much smaller than that for pure filler and was about 4%. This was likely due to the removal of a part adsorbed by nanoSiO_2_ water during the drying of this powder after the modification with DmiBr. The same effect was achieved for nanoSiO_2_/IL20. Ionic liquid present on nanoSiO_2_ surface decomposed in the second step of thermal decomposition in the temperature range of 150–550 °C with a weight loss of approximately 13%. Reducing this value by the weight loss of pure silica in the same range of temperature made it possible to obtain the content of DmiBr on the surface of the modified filler, which was 9%. Thermal decomposition of nanoSiO_2_/IL20 was similar to that of nanoSiO_2_/IL10, with a weight loss in the second step of decomposition of approximately 18%, so that the content of DmiBr that had bonded to the nanoSiO_2_ surface was approximately 15%, and the calculated efficiency of modification was approximately 74% (Table 3). Studying the MS spectra for nanoSiO_2_/IL10, at a temperature above 100 °C, a low intensity band was achieved for m/z ratio 17 and 18 that corresponds to water desorption (Table 4). Above 120 °C, the slow release of C_x_H_y_^+^ and C_x_H_y_N_z_^+^ ions could be seen, suggesting the beginning of DmiBr decomposition on the nanoSiO_2_ surface.

In TG curves corresponding to the thermal decomposition of CB/IL10 and CB/IL20 fillers, a small weight loss at a temperature below 100 °C occurred, approximately 0.3% for CB/IL10 and 0.7% for CB/IL20 (Figure 4). This may result from the desorption of solvent, which remained in the powder after its modification with DmiBr. The weight losses of approximately 8.8% (CB/IL10) and 16.1% (CB/IL20) in the temperature range of 150–550 °C were associated with thermal decomposition of DmiBr. The weight loss observed in this temperature range for pure CB was about 0.2, so the content of ionic liquid on the CB surface was calculated as 8.6% and 15.9% for CB/IL10, and CB/IL20, respectively. Therefore, the efficiency of CB modification with DmiBr was 86% (CB/IL10) and 80% (CB/IL20) (Table 3). The presence of ionic liquid in modified CB powders was confirmed by MS spectra with characteristic m/z ratios for thermal decomposition of DmiBr (Table 4).

The last filler modified with DmiBr was nanosized CaO. On the TG curve corresponding to the thermal decomposition of pure CaO, the first weight loss (1.5%) at a temperature below 150 °C could be observed (Figure 5). It resulted from desorption of moisture; CaO is a hygroscopic powder often used in rubber compounds to absorb moisture. According to MS analysis (Table 4), the next weight loss (20%) in the temperature range of 350–600 °C was accompanied by the release of CO_2_, likely due to the thermal decomposition of precursors used for synthesis of nanosized CaO, such as calcium carbonate or calcium hydroxycarbonate. Moreover, CaO can absorb CO_2_, which is released when heating above the temperature 400 °C [57]. In TG curves corresponding to thermal decomposition of CaO/IL10, three steps were shown. The first at a temperature below 200 °C with a weight loss of 1.6% may result from desorption of moisture or solvent, which remained in the modified powder after modification with DmiBr. The second step of decomposition proceeded in the temperature range of 200–350 °C with a weight loss 6.7%. Such weight loss was not observed in the TG curve of pure CaO, so it corresponds to the decomposition of DmiBr. Moreover, MS spectra showed m/z ratios characteristic for ionic liquid decomposition. The next weight loss of about 17.7%, in the temperature range of 350–600 °C, was associated with thermal decomposition of precursors used for synthesis of nanosized CaO and was accompanied by the release of CO_2_ as was confirmed by MS analysis (m/z 44). In the case of CaO/IL20, similar thermal decomposition was achieved. The weight loss in the temperature below 200 °C (approximately 1.9%) was due to desorption of moisture and solvent after the modification process. The second weight loss in the temperature range of 200–350 °C (12.7%) resulted from the decomposition of DmiBr, whereas the weight loss in the last step of approximately 16.4%, in the temperature range of 350–600 °C, corresponded to thermal decomposition of synthesis precursors, with a release of CO_2_ as confirmed by MS analysis [57]. The efficiency of CaO modification with DmiBr calculated, based on the proper weight losses was 67% (CaO/IL10) and 64% (CaO/IL20), and thus the lowest of all studied fillers. Therefore, it can be concluded that the surface of nanosized CaO exhibited the lowest reactivity with DmiBr. This may be a result of the high content of CaO synthesis precursors that act as impurities reducing the reactivity of the functional groups present on the surface of this filler or reducing their availability to interaction with DmiBr.

In summary, the results of TG analysis made it possible to identify the weight loss associated with thermal decomposition of DmiBr in modified fillers, thus confirming the presence of ionic liquid and the effectiveness of modification. MS analysis of evolved gases was very helpful in predicting the course of DmiBr thermal decomposition and thermal stability of modified fillers. Based on the weight loss associated with decomposition of DmiBr in modified fillers, it was possible to calculate the efficiency of modification and compare the surface reactivity of studied fillers toward DmiBr. Regarding the efficiency of modification, silica VN3 and CB exhibited similar ability for interaction with DmiBr. More than 80% of the initial amount of DmiBr was bonded to the surface of these fillers in the modification process. The efficiency of modification for nanoSiO_2_/IL10 was significantly higher (92%) than for nanoSiO_2_/IL20 (74%). Probably 10% of DmiBr relative to the amount of used silica was enough to cover almost the entire surface of this filler, and there were no functional groups on the nanoSiO_2_ surface available for interaction with the DmiBr. In this case, the excess of DmiBr remained in acetone due to favorable hydrogen-bonding interactions between this solvent and IL [54,58]. This was also confirmed by extending the time of solvent evaporation, at the same temperature and pressure, from 30 min. for pure acetone to 250 min. for acetone after fillers modification with DmiBr. As previously mentioned, nanosized CaO showed the lowest reactivity with DmiBr most likely due to the presence of impurities in the form of residual synthesis precursors.

### 3.2. Differential Scanning Calorimetry (DSC)

DSC analysis was employed to study the thermal behaviour of DmiBr, pure and DmiBr-modified fillers. The use of this method was also intended to examine if the ionic liquid is permanently attached to the surface of fillers or whether it desorbs at a specific temperature. Further, assuming that the aim of filler modification with DmiBr is to improve their dispersion in the elastomer, this ionic liquid should remain bound to the filler surface at the vulcanization temperature of rubber compounds, which is usually in the range of 140–180 °C depending on the type of rubber and curing system used.

**Figure 6:**
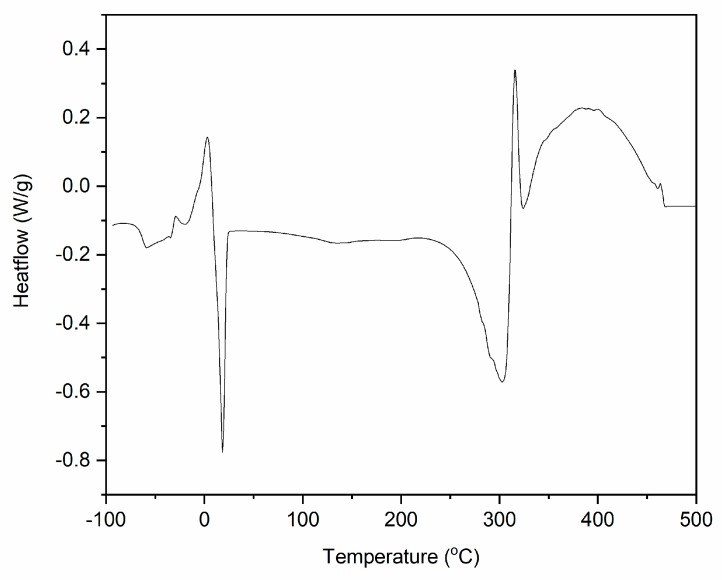
Differential Scanning Calorimetry **(**DSC) curve for DmiBr (exo^).

**Figure 7:**
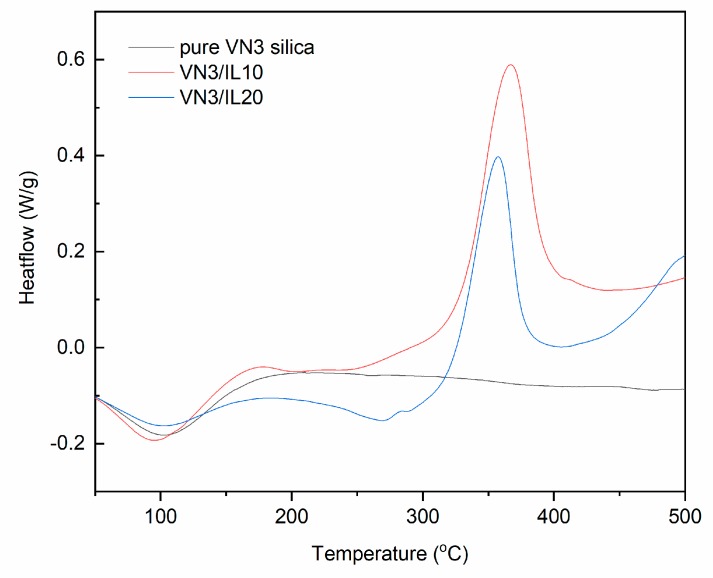
DSC curves for pure and DmiBr-modified VN3 silica (exo^).

**Figure 8:**
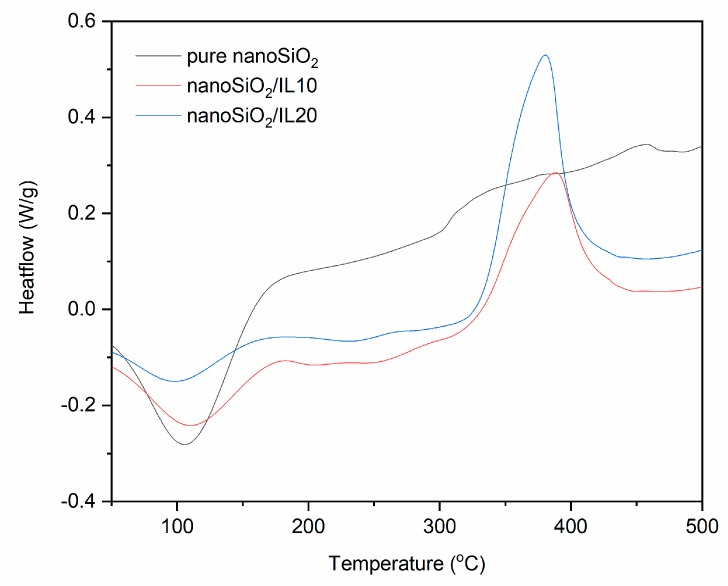
DSC curves for pure and DmiBr-modified nanoSiO_2_ (exo^).

**Figure 9:**
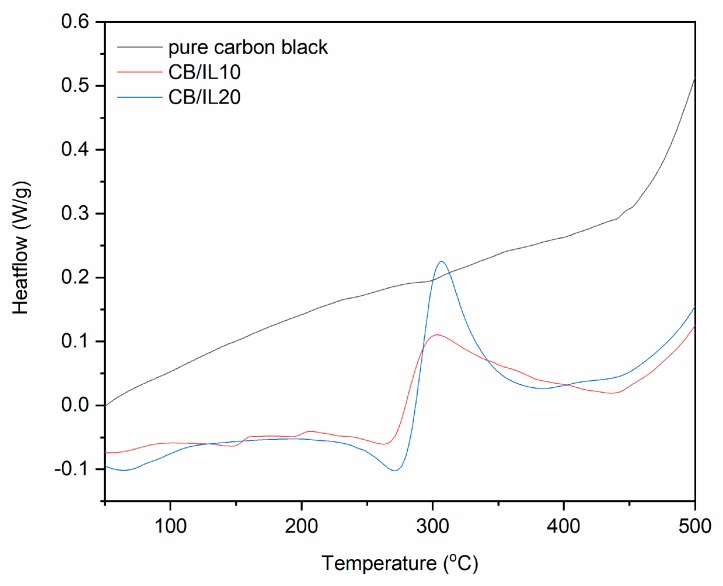
DSC curves for pure and DmiBr-modifed CB (exo^).

**Figure 10:**
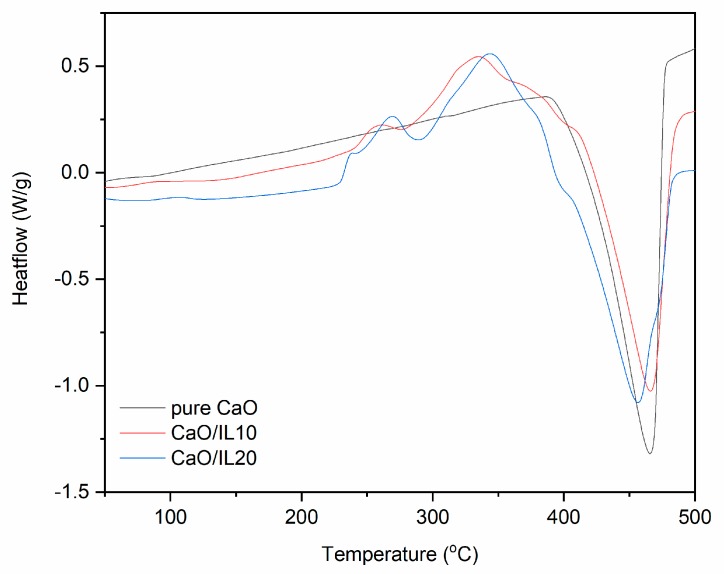
DSC curves for pure and DmiBr-modified nanosized CaO (exo^).

DSC results for DmiBr are presented in Figure 6 and Table 5, whereas DSC curves for pure and DmiBr-modified fillers are shown in Figure 7, Figure 8, Figure 9 and Figure 10 and presented in Table 6.

DmiBr showed a glass transition with T_g_ at approximately −63 °C. Next, cold crystallization occurred at a temperature 18.2 °C, followed by the melting of the crystalline phase at 3.2 °C. Thermal decomposition of DmiBr started in the temperature above 200 °C. First, an endothermic peak with a temperature of 300 °C was observed on the DSC curve, and then the process changed its character to exothermic at a temperature of 305 °C. Similar stages of thermal decomposition could be seen on DSC curves of DmiBr-modified fillers. The first endothermic step is desorption of DmiBr from the surface of filler accompanied with its partial decomposition (probably fragmentation of dodecyl chain and the departure of bromide anion), and the second one is further exothermic decomposition of the DmiBr (mainly imidazolium ring of ionic liquid into different fragments, as was confirmed by TG-MS). Similar thermal behaviour was observed for other imidazolium ILs [59,60]. 

Wide endothermic peaks could be observed in the DSC curves for pure and DmiBr-modified VN3 silica (Figure 7). The temperature ranges of these peaks were as follows: 60–113 °C (pure VN3); 33–123 °C (VN3/IL10); 39–124 °C (VN3/IL20). These peaks correspond to the first weight loss in TG curves and resulted from desorption of moisture. In the case of DmiBr-modified VN3, it could also be the desorption of acetone remaining after the modification process. Above a temperature of 160 °C, the baseline did not change considerably for pure silica until the end of the measurement, whereas for modified powders, an endothermic peak appeared in the temperature range of 229–309 °C, corresponding with the second weight loss in the TG curves. This was due to the desorption of DmiBr from the surface of fillers. However, taking into account the results of TG-MS, the process of DmiBr desorption is accompanied with its partial decomposition, since in temperatures above 200 °C, the slow release of C_x_H_y_^+^ and C_x_H_y_N_z_^+^ ions could be seen, suggesting the beginning of DmiBr decomposition (Table 4). After desorption, DmiBr decomposed exothermically above the temperature 320 °C. Most importantly, DmiBr was remaining on the VN3 silica surface in the range of the elastomer vulcanization temperature.

Similar thermal behavior was observed for DmiBr-modified nanoSiO_2_ (Figure 8). Desorption of moisture, accompanied by desorption of solvent for modified powders, occurred in the temperature range of 52–162 °C (pure nanoSiO_2_), 56–111 °C (nanoSiO_2_/IL10), and 49–155 °C (nanoSiO_2_/IL20). The process of DmiBr desorption from the surface of modified filler, accompanied with its partial decomposition, started at 187 °C for nanoSiO_2_/IL10 and 196 °C for nanoSiO_2_/IL20, i.e., at a lower temperature than that of VN3 silica. Next, an exothermic peak of DmiBr thermal decomposition appeared above 330 °C. Regarding the range of commonly used vulcanization temperatures 140–180 °C, DmiBr-modified nanoSiO_2_ should also be stable during processing of rubber compounds.

On the DSC curve of CB/IL10, a broad endothermic peak at a temperature range of 29–87 °C can be observed (Figure 9) most likely as a result of inaccurate evaporation of the solvent and corresponding to the first weight loss on the TG curve presented earlier. Next, the endothermic process could be seen from 220 to 275 °C, which was associated with desorption of DmiBr and beginning of its thermal decomposition, as was confirmed by TG-MS analysis. However, in the case of CB ionic liquid should also not desorb from the surface of filler during elastomer vulcanization. After desorption, DmiBr decomposed in the temperature range of 276–384 °C. Similar thermal effects were achieved for CB/IL20. Desorption of DmiBr from the CB surface with the beginning of its decomposition proceeded in a temperature range similar to the VN3 silica.

Regarding the DSC curve for pure nanosized CaO, no thermal effects were observed in the temperature range of 25–400 °C, whereas at a higher temperature, an endothermic peak appeared corresponding to the last weight loss on the TG curve (Figure 10). Therefore, it was concluded that this is a thermal decomposition of CaO precursors, which remained after synthesis of this filler. Similar peaks were achieved for DmiBr-modified CaO. A wide endothermic peak in the temperature range of 35–260 °C occurred for CaO/IL10. This may be due to desorption of solvent after modification accompanied by desorption of DmiBr. Therefore, it could be expected that DmiBr will desorb from the CaO surface during vulcanization of rubber compounds. After desorption, ionic liquid decomposed in the temperature range of 290–402 °C. The same thermal effects were observed for CaO/IL20. The first endothermic peak was in the temperature range of 33–259 °C. The heat of the first peak for CaO/IL20 (37 kJ/mole IL) was almost twice as large as for CaO/IL10 (22 kJ/mole IL), which may confirm the assumption that this peak corresponded to the desorption of DmiBr from the CaO surface. After desorption, DmiBr decomposed exothermically in the temperature range of 296–387 °C. It should be noted that in the case of CaO, desorption of DmiBr proceeded in the lowest temperature in comparison with the other fillers. This confirmed the lowest reactivity of CaO with DmiBr and weak interaction with this ionic liquid.

Analyzing the enthalpies of DmiBr desorption from the surface of fillers accompanied with its thermal decomposition (Table 6), it should be noted that the values of enthalpy determined for fillers modified with 20 wt.% of DmiBr are significantly higher compared to the fillers modified with 10 wt.% of DmiBr. This correlates with the higher content of DmiBr in these fillers.

Summarizing, the DSC results of DmiBr-modified fillers are fully compatible with the TG results and allowed for the identification and confirmation of the nature of the thermal transitions occurring on TG curves. Moreover, DSC made it possible to determine the temperature of DmiBr desorption from the surface of modified fillers and consequently their thermal stability in the vulcanization temperature of rubber compounds.

### 3.3. Scanning Electron Microscopy (SEM)

**Figure 11:**
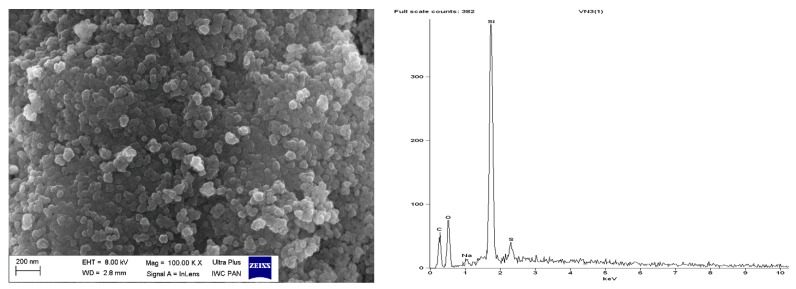
Scanning electron microscopy (SEM) with Energy-dispersive X-ray spectroscopy (EDS) analysis for pure VN3 silica.

**Figure 12:**
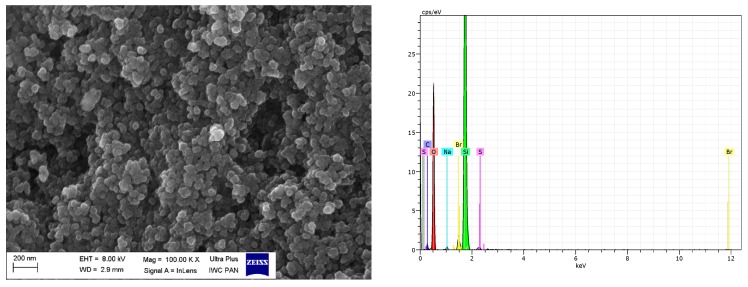
SEM with EDS analysis for VN3/IL20.

**Figure 13:**
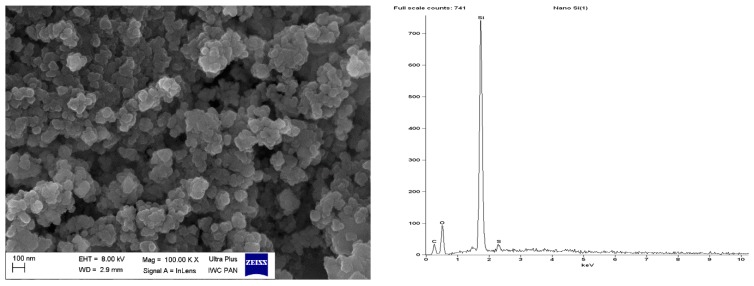
SEM with EDS analysis for pure nanoSiO_2_ silica.

**Figure 14:**
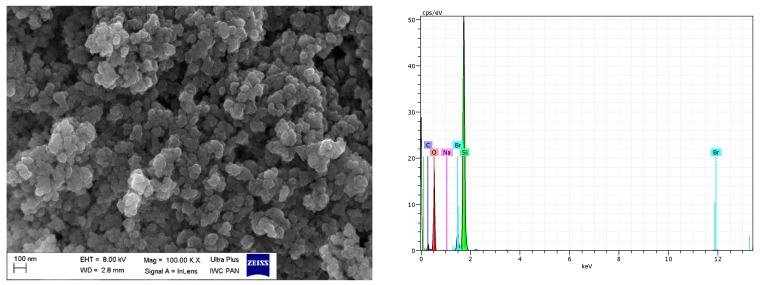
SEM with EDS analysis for nanoSiO_2_/IL20.

**Figure 15:**
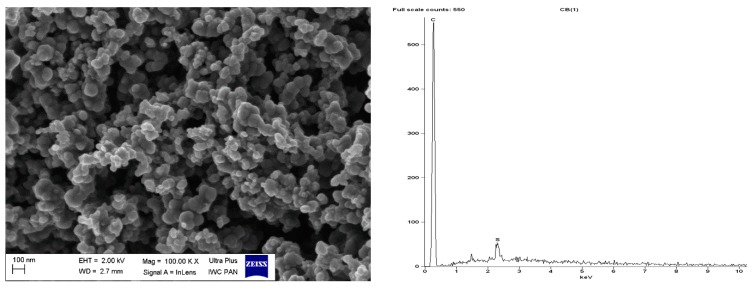
SEM with EDS analysis for pure CB.

**Figure 16:**
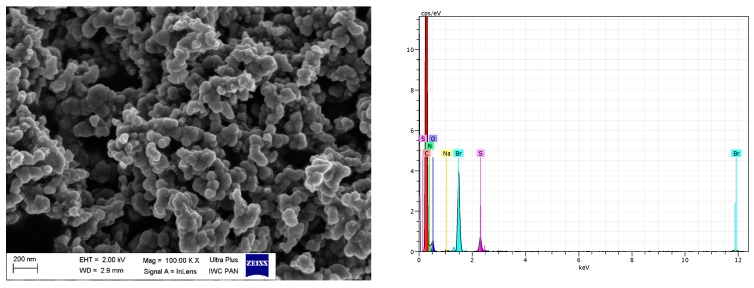
SEM with EDS analysis for CB/IL20.

SEM images were taken to study the particle morphology and size of DmiBr-modified fillers. Additionally, EDS analysis was employed to confirm the presence of DmiBr on their surface. The exemplary results for pure fillers and modified with 20 wt.% of DmiBr are presented in Figure 11, Figure 12, Figure 13, Figure 14, Figure 15 and Figure 16 and summarized in Table 7.

SEM images showed that primary particles of pure and DmiBr-modified VN3 and nanoSiO_2_ exhibit spherical morphology and average size of approximately 100 nm (Figure 11, Figure 12, Figure 13 and Figure 14). Therefore, immobilization of IL on the surface of these fillers did not significantly affect the size and morphology of particles. EDS spectra confirmed the presence of DmiBr on VN3 and nanoSiO_2_ surfaces (bands for C and Br). Moreover, the wt.% content of Br element in EDS spectra of VN3/IL20 and nanoSiO_2_/IL20 is more than twice as large as for samples VN3/IL10 and nanoSiO_2_/IL10, which corresponds to amount of DmiBr used to modify these fillers (20 wt.% and 10 wt.% respectively). The wt.% content of C element in the EDS spectra was also higher for fillers modified with 20 wt.% of DmiBr. No bands for Br in the EDS spectrum of pure VN3 and nanoSiO_2_ were observed, which confirms that the presence of bromine in DmiBr-modified fillers is due to the presence of an ionic liquid. The C bands in the EDS spectra of pure silicas results from the coating of their surface with a layer of carbon before the measurement.

Regarding DmiBr-modified CB, this filler consists of fine, varied in size, spherical and elongated primary particles. Most of the CB particles are 100–200 nm in size, but smaller particles less than 100 nm in size could be seen in the SEM images (Figure 16). Pure CB consists of particles of similar size and morphology. EDS analysis confirmed the presence of DmiBr on the modified CB surface. Apart from the C band in the EDS spectra, which could also result from CB, additional bands for N and Br were identified. Such bands were not observed in the EDS spectra of pure CB (Figure 15).

**Figure 17:**
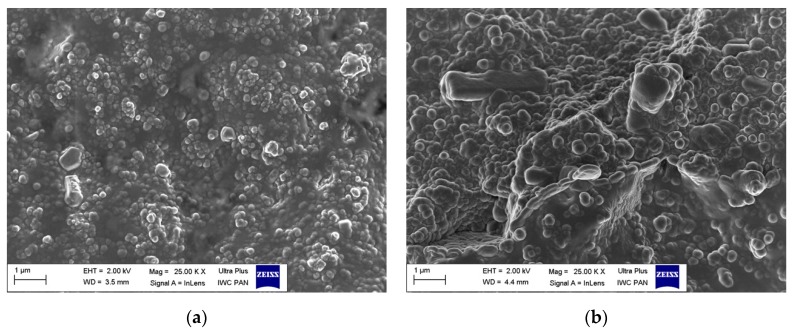
SEM images of vulcanizates containing: (**a**) VN3/IL20; (**b**) CB/IL20.

To study the behavior of DmiBr-modified fillers in the rubber matrix, SEM images were taken for the fractures of vulcanizates based on ethylene-propylene-diene elastomer (EPDM). SEM images for vulcanizates, containing VN3/IL20 and CB/IL20 are presented as examples (Figure 17). It can be seen that the particles of DmiBr-modified fillers were quite homogeneously dispersed in the elastomer matrix and exhibited good adhesion to the elastomer.

## 4. Conclusions

The presented results confirmed the very important role of thermal analysis and SEM in studying the efficiency of filler modification, with ionic liquid dissolved in acetone. Such modified fillers could be applied to improve the physico-chemical properties of elastomers.

TG/MS and SEM/EDS analysis were successfully employed to confirm the presence of ionic liquid in modified fillers and the effectiveness of the modification. By identifying the weight loss associated with the thermal decomposition of DmiBr in modified fillers, it was possible to calculate the efficiency of their modification and compare the surface reactivity of studied fillers with DmiBr. It could be concluded that silica VN3 and CB exhibited a high and comparable ability for the interaction with DmiBr, since the efficiency of modification was higher than 80% for both fillers. Regarding nanosized silica, the efficiency of modification was significantly higher for nanoSiO_2_/IL10 than for nanoSiO_2_/IL20. Probably 10% of DmiBr, relative to the amount of used silica, was sufficient to cover almost the entire surface of this filler. Nanosized CaO showed the lowest reactivity with DmiBr, due to the presence of residual synthesis precursors, which could reduce the availability of CaO active groups for interaction with ionic liquid.

DSC fully completed the TG results and made it possible to identify and determine the nature of the thermal effects occurring on the TG curves. Furthermore, using this method, it was possible to study the temperature of DmiBr desorption, from the surface of modified fillers, and consequently their thermal stability in the vulcanization temperature of rubber compounds, which is crucial for improving the dispersion of fillers in elastomer matrix. The lowest desorption temperature of DmiBr was determined for modified CaO, which affirmed the poor reactivity of this filler to DmiBr.

SEM analysis confirmed that DmiBr-modified fillers are homogeneously dispersed in the EPDM matrix and exhibited a good adhesion to the elastomer. Therefore, further research will be carried out on the immobilization of ILs with other cations, e.g. pyridinium, piperidinium, or pyrrolidinium salts. The influence of the ILs anion should also be investigated. Future studies will focus on determining the factors responsible for the different activity of ILs in the process of fillers modification, and on investigating the type of interactions between ILs and different fillers. Finally, the influence of ILs immobilized on the surface of fillers on the vulcanization of rubber compounds and physico-chemical properties of vulcanizates will be studied. 

## Figures and Tables

**Table 1:** Weight losses (Δm) obtained from thermogravimetric (TG) curves for pure and DmiBr-modified fillers.

**Sample**	**Δm < 150 °C (%)**		**Δm > 150 °C (%)**	**Residue at 600 °C (%)**
DmiBr	3.5		96.1	0.4
VN3	3.7		2.2	94.1
VN3/IL10	5.3		10.7	84.0
VN3/IL20	2.5		17.9	79.6
nanoSiO_2_	10.0		3.2	86.8
nanoSiO_2_/IL10	4.0		12.4	83.6
nanoSiO_2_/IL20	2.6		17.9	79.5
CB	-		0.2	99.8
CB/IL10	0.3		8.8	90.9
CB/IL20	0.7		16.1	83.2
**Sample**	**Δm < 200 °C (%)**	**Δm 200–350 °C (%)**	**Δm > 350 °C (%)**	**Residue at 600 °C (%)**
CaO	1.5	-	20.0	78.5
CaO/IL10	1.6	6.7	17.7	74.0
CaO/IL20	1.9	12.7	16.4	69.0

**Table 2:** Mass Spectrometry (MS) analysis of DmiBr.

Mass/Charge Ratios of Secondary Ions for DmiBr		
>180 °C	>240 °C	>360 °C
12, 13-C^+^; 15-CH_3_^+^; 39-C_3_H_3_^+^, 42-C_3_H_6_^+^; 51-C_4_H_3_^+^, C_3_NH^+^; 52-C_4_H_4_^+^, C_3_NH_2_^+^; 79, 80, 81-Br	14 – CH_2_^+^; 27 – C_2_H_3_^+^; 30 – C_2_H_6_^+^; 42 – C_3_H_6_^+^; 43 – C_3_H_7_^+^; 55 – C_4_H_7_^+^, C_3_NH_5_^+^; 56 – C_4_H_8_^+^, C_3_NH_6_^+^	12 – C^+^; 26 – C_2_H_2_^+^; 27– C_2_H_3_^+^; 41– C_3_H_5_^+^; 42 – C_3_H_6_^+^; 43 – C_3_H_7_^+^

**Table 3:** DmiBr content in modified fillers and efficiencies of modification.

Sample	Amount of used DmiBr (mmole/g of filler)	Amount of DmiBr in Modified Filler (mmole/g of filler)	Efficiency of Modification (%)
VN3/IL10	0.33	0.28	85
VN3/IL20	0.66	0.52	79
nanoSiO2/IL10	0.33	0.30	92
nanoSiO2/IL20	0.66	0.49	74
CB/IL10	0.33	0.28	86
CB/IL20	0.66	0.53	80
CaO/IL10	0.33	0.22	67
CaO/IL20	0.66	0.42	64

**Table 4:** MS analysis for DmiBr-modified fillers.

**m/z of Secondary Ions for DmiBr-Modified VN3**			
Sample	120–200 °C	>200 °C	>400 °C
VN3/IL10	26, 48, 64	12, 13, 15, 18, 26, 27, 35, 79, 80	12, 18, 26, 44, 46, 48
VN3/IL20	12, 29, 31, 52	12, 15, 29, 31, 35, 47, 50, 79, 80, 81	12, 29, 50, 52
**m/z of Secondary Ions for DmiBr-Modified NanoSiO_2_**			
nanoSiO_2_/IL10	12, 17, 18, 24, 25, 26, 27, 60, 62	63, 64, 72, 78, 79, 80	12, 15, 17, 18, 24, 25, 26, 27, 30, 48, 60, 63, 64, 72, 78, 79, 80
nanoSiO_2_/IL20	12, 15, 29, 26, 30	12, 15, 26, 29, 30	12, 26, 29, 30
**m/z of Secondary Ions for DmiBr-Modified CB**			
Sample	160–200 °C		>200 °C
CB/IL10	78		12, 13, 14, 15, 47, 79, 80
CB/IL20	18, 47		12, 13, 15, 18, 47, 79, 80
**m/z of Secondary Ions for DmiBr-Modified CaO**			
Sample	160–200 °C	>200 °C	>300 °C
CaO/IL10	12, 14, 15, 44, 45	15, 31, 35, 44, 47, 50, 52, 79, 80	12, 15, 30, 44
CaO/IL20	-	15, 31, 35, 39, 47, 48, 50, 52, 79, 80	12, 15, 44

**Table 5:** Differential Scanning Calorimetry **(**DSC) analysis of DmiBr (T_g_—glass transition, T_cc_—cold crystallization, T_m_—melting, T_dec_—decomposition temperatures, ΔH—enthalpy of phase transitions).

T_g_ (°C)	T_cc_ (°C)	ΔH_cc_ (kJ/mole IL)	T_m_ (°C)	ΔH_m_ (kJ/mole IL)	T_dec (endo)_ (°C)	ΔH_dec (endo)_ (kJ/mole IL)	T_dec (exo)_ (°C)	ΔH_dec (exo)_ (kJ/mole IL)
−63.0	18.2	−16.3	3.2	16.5	299.6	−44.0	317.1	125.5

**Table 6:** DSC analysis for pure and modified fillers.

Sample	Desorption of Moisture/Solvent (°C)	Desorption/Decomposition of IL Immobilized on the Filler’s Surface		Decomposition of IL	
		Temperature (°C)	Enthalpy (kJ/mole IL)	Temperature (°C)	Enthalpy (kJ/mole IL)
pure VN3 silica	60–113	-	-	-	-
VN3/IL10	33–123	231–309	−5.5	330–392	80.1
VN3/IL20	39–124	229–287	−11.5	322–377	71.2
pure nanoSiO_2_	52–162	-	-	-	-
nanoSiO_2_/IL10	56–111	187–284	−6.0	332–434	57.0
nanoSiO_2_/IL20	49–155	196–321	−7.1	334–403	84.4
pure CB	-	-	-	-	-
CB/IL10	29–87	223–275	−4.0	276–384	33.9
CB/IL20	30–106	237–284	−10.9	286–344	38.9
pure CaO	-	-	-	-	-
CaO/IL10	-	35–260	–22.0	290–402	54.9
CaO/IL20	-	33–259	–36.7	296–387	58.6

**Table 7:** Energy-dispersive X-ray spectroscopy (EDS) analysis of pure and DmiBr-modified fillers.

Filler	Element (wt.%)							
	O K	Si K	C K	Br K	N K	Na K	S K	Total
pure VN3	37.69	18.80	40.81	-	-	0.96	1.75	100
VN3/IL10	50.74	38.72	8.11	1.50	-	0.53	0.41	100
VN3/IL20	52.13	28.55	15.20	3.02	-	0.58	0.53	100
pure nanoSiO_2_	37.43	30.22	31.08	-	-	-	1.27	100
nanoSiO_2_/IL10	51.59	34.89	11.19	2.12	-	-	0.20	100
nanoSiO_2_/IL20	47.91	34.35	13.16	4.50	-	0.08	-	100
pure CB	2.38	-	95.78	-	-	-	4.22	100
CB/IL10	4.63	-	84.88	1.26	8.42	0.12	0.69	100
CB/IL20	3.59	-	85.48	3.43	6.77	0.09	0.64	100
